# Towards the Augmentation of Digital Twin Performance

**DOI:** 10.3390/s23229248

**Published:** 2023-11-17

**Authors:** Quentin Charrier, Nisar Hakam, Khaled Benfriha, Vincent Meyrueis, Cyril Liotard, Abdel-Hakim Bouzid, Améziane Aoussat

**Affiliations:** 1 Arts et Métiers Institute of Technology (AMIT), 75013 Paris, France; nisar.hakam@ensam.eu (N.H.);; 2 ERM Automatismes, 84200 Carpentras, France; 3 École de Technologie Supérieure, University of Montreal, Montreal, QC H3C 1K3, Canada

**Keywords:** Industry 4.0, sensors, Digital Twin, analysis

## Abstract

Digital Twin (DT) aims to provide industrial companies with an interface to visualize, analyze, and simulate the production process, improving overall performance. This paper proposes to extend existing DT by adding a complementary methodology to make it suitable for process supervision. To implement our methodology, we introduce a novel framework that identifies, collects, and analyses data from the production system, enhancing DT functionalities. In our case study, we implemented Key Performance Indicators (KPIs) in the immersive environment to monitor physical processes through cyber representation. First, a review of the Digital Twin (DT) allows us to understand the status of the existing methodologies as well as the problem of data contextualization in recent years. Based on this review, performance data in Cyber–Physical Systems (CPS) are identified, localized, and processed to generate indicators for monitoring machine and production line performance through DT. Finally, a discussion reveals the difficulties of integration and the possibilities to respond to other major industrial challenges, like predictive maintenance.

## 1. Introduction

The new framework of Industry 4.0, known as Reference Architecture Model Industry 4.0 (RAMI 4.0), has an objective of fully digitizing the industrial shop floor, according to the German committees DIN and DEK [1]. Thus, the DT applications become a popular application to implement in the cyber representation of an industrial system [2]. This surge is related to the deployment of CPS [3], Internet-of-Things (IoT) [4], and cloud-based services [5]. They allow us to integrate the notion of digitization, real-time monitoring, modularity, and interoperability, according to El-Zant et al. [6]. In addition, the DT tasks are also correlated with predictive maintenance [7] and dynamic operation scheduling [8] that take advantage of Machine Learning (ML) and Deep Learning (DL).

However, this transformation is still under development since no standardized methodology that responds to all properties is verified to integrate the full potential of DT, according to the review of the next section. Our objective is to analyze the missing steps for a full DT framework and to introduce a standard framework that allows a simple and effective implementation of performance indicators implementation inside the cyberworld.

## 2. Digital Twin

The fourth industrial revolution is a term that has become popular throughout various branches such as healthcare [9] and manufacturing [10]. This evolution is coupled with the digitization that served as a base for the surge of DT in the industry. 

### 2.1. Data Availability

Digitization requires a sensory detection system to create coherence between the physical entity and the cyber world. This requirement, also known as a “data-driven approach”, allows control of the production line and to assure an ongoing production cycle. Permulla et al. studied the DEEP-Cyberia program [11]. They found that data collection and performance analysis are key factors in building the connection of physical and virtual. 

Kalstoom et al. provided a systematic review of the RAMI4.0 [4]. It is revealed that Internet-of-Things (IoT) is an interesting domain among researchers since it helps to remotely monitor and control an application. The authors conclude their paper with four essential questions: “What are cost-effective methods to capture data?”, “How can the use of power be optimized?”, “How can we manage IoT data?”, and “How can IoT develop manufacturing lines?”.

Christou et al. developed a Quantitative Association of Rule Mining Algorithm (QARMA) [12]. The algorithm serves as a method to extract rules, enhancing the support-confidence of a system. However, the authors reveal that the efficiency of QARMA depends on the data collected. These data are still not fully exploitable since data only refers to basic functions and not to general performance data, such as vibration, acoustic, and current.

The two reviews as well as the QARMA study focus on a lack of data elaboration for performance on the industrial shop floor. Simultaneously, the surge of DT requires these data to create coherence with real-life systems. This importance is presented in the section below. 

### 2.2. Digital Twin Technologies

With respect to Permulla et al. [11] and Kalstoom et al. [4], sensor technology is an important technology to equip the systems for performance supervision. However, the control of a production line is done through a third-party control unit, respecting the industrial norms, according to Negri et al. [13]. This later control feedback is the study for another aspect of DT, which is known as decision classification from DT, introduced by Tao et al. [14]. During our implementation, we address the problem of sensor selection, configuration, and installation.

### 2.3. Methodologies for Digital Twin

**Figure 1:**
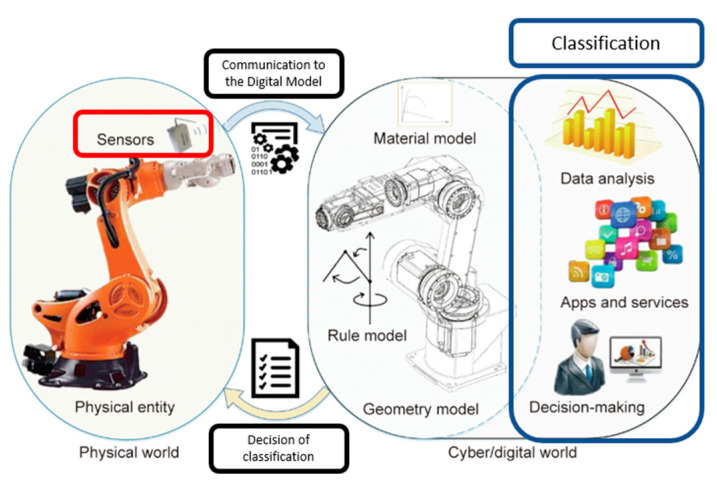
Correlation of the physical and cyber world.

The DT uses the 3D model of the equipment to mimic the behavior of the actual module through collected data. These data are transformed into metadata to allow visualization inside the cyber world of the DT. According to Tao et al., CPS and DT have a similar objective, which is the representation of a system in a cyber world [14] (refer to Figure 1).

Tao et al. presented in their review/comparison paper, the difference between the CPS and DT [14]. It is concluded that CPS emphasizes data extraction from sensors and control signals through remote actuators, while DT is used to handle data structures, perform advanced analysis, and guide operators on the production performance. They also describe the decision-making process in the DT as preliminary since the integration of IoT is still at preliminary stages in the industry, due to financial and innovative reasons.

**Figure 2:**
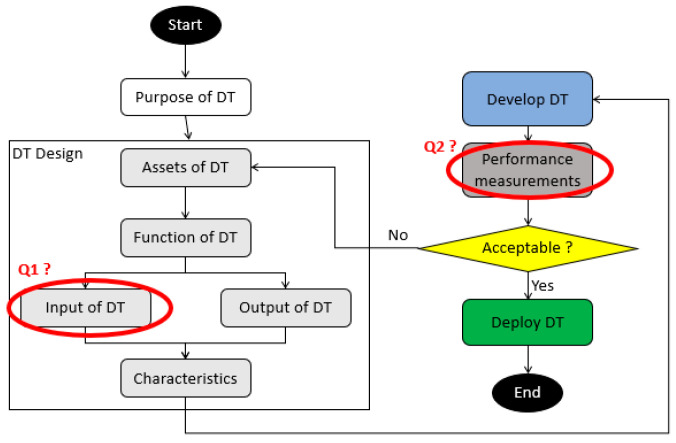
Standard conception of DT with problematics.

In 2023, Psarommatis et May revised a total of 169 papers and found a gap in the standard for designing a DT [15]. They suggested a standard methodology that goes on from defining the purpose of DT, designing the DT, testing it, and then evaluating it before deployment, refer to Figure 2.

The conception steps are organized to analyze the existing data, such as transferability and effectiveness. These data are divided into inputs, like positioning, vibration, and energy signals, and outputs, such as a control signal to activate an operation. However, the future perspectives of Psarommatis et May (2023) are the collection of performance data, refer to Q1 inside Figure 2, and the development of performance measurements, refer to Q2 inside Figure 2, to validate the conception approach and to deployment of the DT in a production system.

### 2.4. Observation

The review of Psarommatis et May (2023) and the architecture of Tao et al. (2019) reveal that the DT has become the interest of Industries. Both papers revise a total of 228 papers where the majority identify the data availability and performance measurements as the main sources for the successful deployment of DT. All the articles, cited in this paper or cross-referenced with the review papers, have helped to position our work. Indeed, the directions taken are consistent with their perspectives cited. The aim is to complement existing frameworks by automating the collection, processing, and presentation of performance data in DT. Simultaneously, they identify these fields as the major deficit nowadays, since industrial sensors are costly, and the integration is not straightforward. They require to adapt the infrastructure, manage the cable installation, control the latency of a network, and configure mitigation of data to a high-performance desktop, located at a cloud computing provider. Thus, in our paper, we are not only presenting cost-effective ways to measure performance data but also identifying a framework that allows thcreation of performance indicators inside the DT based on these process data and a local edge/fog computing algorithm.

## 3. Methodology for KPI in Digital Twin

**Figure 3:**
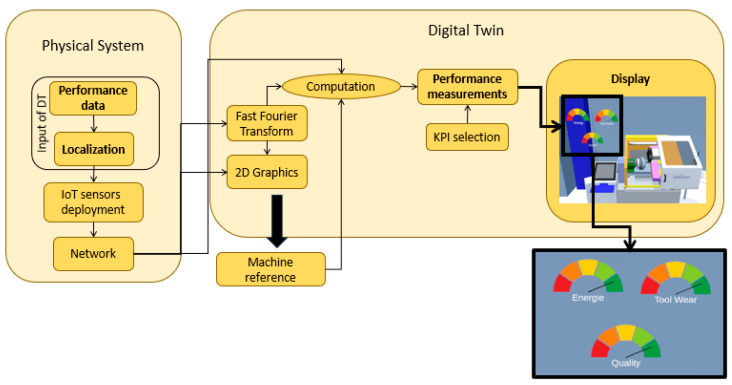
Methodology for KPI integration.

KPIs, also known as “Key Performance Indicators”, are simple representations for operators to analyze the behavior of a system. Our objective is to find some KPIs to visualize in the DT, allowing us to understand the latency of connection and to monitor the behavior of systems. This integration can respond to the requirements for the identification of performance measurement tools for DT shown in Figure 2. As a result, we designed a methodology that responds to the three questions of Psarommatis et May (2023): “Which data?”, “What to compute?”, and “How to display?”, as shown in Figure 3.

Most systems only inform about internal data of systems, also known as operational data. However, these data serve as a feedback loop for the operation executed by the system. These operational data do not contribute to the performance analysis since the main objective is only to control the process and not to analyze the performance. 

After discussion with machinist experts, it is concluded that important data for performance lies in the environmental data of the process, like vibration, acoustic emissions, temperature, current, voltage dips, magnetic flux, etc. During conception, most vendors do not consider the extraction of performance data, creating a major deficit for digitization and DT development. IoT sensors fill this gap by making it possible to add a sensory layer to every system. This layer provides the opportunity to extract the performance data relevant to our case. 

After defining the performance data of a machine and their respective measurement place, a further study is conducted to determine the characteristics of IoT sensors. These sensors are either bought as plug-and-play modules or assembled through a development kit. For example, the Bosch XDK110, which is an IoT board, was developed to fit eight sensors for maximum data extraction, such as a magnetometer, a light sensor, a gyroscope, an accelerometer, a temperature sensor, a noise sensor, a humidity sensor, and a pressor sensor. This board is designed to attach on the part to monitor, connecting wirelessly to a cloud platform [16] or to a local server and presents the possibility for extension through external modules. However, this latter option requires a reconfiguration of the firmware as well as an adaptation of data flow in the data transfer pipeline. The other approach is a self-developed board using the Espressif development kit. In addition, it is configured for local or cloud deployment on a selected system. These steps conclude with a known problem in Infrastructure Technology (IT), which is the latency in connection. Since most IoT sensors are wirelessly connected, the latency increases with the number of devices on a network. This concept is proven by different research presented in the Section 4.

### Design of Digital Twin

**Figure 4:**
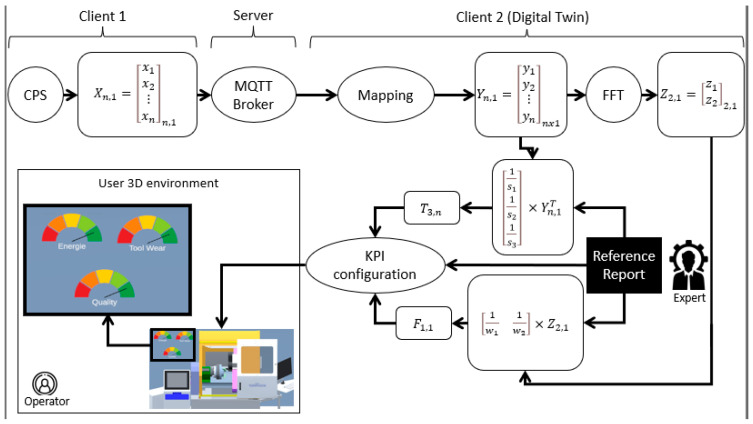
Pipeline for data treatment and KPI configuration.

According to the literature review of Section 2, data availability is only one challenge for DT design. Another one is the definition of performance indicators to measure the coherence between physical and cyber worlds. In this section, we propose a mathematical and engineering pipeline, allowing us to transform the raw data into significant KPI, presented in Figure 4.

First, the transfer protocol is defined. D’Ortona et al. used the Message Queue Telemetry Transport (MQTT) protocol to send data around the framework of smart cities [17]. They tested the protocol on a small scale and deduced that through a wireless connection, the MQTT protocol performed at the remarkable delay of 58 milliseconds, putting in the forefront of the 4G technology. As a result, we thought that MQTT is beneficial for our approach, where the broker runs as a service on a desktop, for example, the desktop of DT. In addition, Figure 4 presents two clients: client 1 is the IoT sensor, installed on the physical system as described previously, and client 2 is the DT, receiving all these data for performance measurement.

Second, the pipeline differentiates between two operators: an expert and an operator. The expert represents the maintenance engineer with a deep knowledge of system behavior, while the operator is a system user with minimal training to use the equipment. Our method formulates a reference report, based on expert knowledge, and uses mathematical models to obtain binary vectors, represented in KPIs.

Third, the vectors, presented in Figure 4, are defined in Table 1.

These vectors are defined in our pipeline to represent a systematical approach to transform the raw time signal into physical values through a mapping equivalency, shown in the mapping block of the pipeline and presented mathematically in Equation (1).

$$4095\;\to IoT_{max}$$

$$x_{t}\;\to \;y_{t}$$

$$0\;\to {IoT}_{min}$$

(1) $$y_{t}=\frac{x_{t}}{4095}*\left(IoT_{max}-IoT_{min}\right)+IoT_{min}$$

where $x_{t}$ represents the measured sample by the sensor, $IoT_{min}$ represents the minimum physical measurable value by the sensor, $IoT_{max}$ represents the maximum physical measurable value by the sensor, and $y_{t}$ represents one entry of the mapped vector $Y_{n,1}$. The values 0 and 4095 are obtained since most Analog to Digital Converter (ADC) are 16-bits. 

The Y vector is considered as an input to the DT, shown in Figure 3. However, the physical performance is not only determined by the time series but also obtained from the frequency signal. This step is represented by a Fast Fourier Transform (FFT), refer to Figure 3 and Figure 4. This transformation computes different properties, our focus is on the fundamental frequency and the percentage of harmonics. This block is represented mathematically by Equation (2).

(2) $$z_{t}=\sum_{p=0}^{n-1}y_{p}*e^{-\frac{2\pi ipk}{n}}=\sum_{p=0}^{\frac{n}{2}-1}y_{p}^{even}*e^{-\frac{2\pi i\left(2p\right)k}{n}}+\sum_{p=0}^{\frac{n}{2}-1}y_{p}^{odd}*e^{-\frac{2\pi i\left(2p+1\right)k}{n}}$$

where the first section represents the Discrete Fourier Transform (DFT) and the second section represents the FFT. The output vector $z_{t}$ is going to be of size n by 1, representing all the frequencies in the signal $y_{t}$. In our pipeline, the Z vector is constructed from 2 values:

$$Z=\left[\begin{array}{c} z_{1}\\ z_{2} \end{array}\right],\;where\;$$

$$z_{1}=\max\left(amplitude\left(z_{t}\right)\right)$$

$$z_{2}=\frac{\sum_{p=0}^{n}{amplitude(z}_{p})}{n},\;if\;z_{p}\;!=fundamental\;frequency$$

Thus $z_{1}$ represents the fundamental frequency and $z_{2}$ represents the percentage of harmonics in the signal.

Fourth, once the vectors Y and Z are computed, a reference report is constructed with the knowledge of a maintenance engineer for the system. This report includes average values for performance signals, maximum/minimum values, optimal frequency for signals, error values, and false correlations. In our dataflow, we used 3 constants to identify optimal time series signals and 2 constants to check the frequencies of the signal. The inverse of these constants is multiplied by the respective vectors, and then conditioned. The binary vectors T and F are used to build the KPIs.

Finally, our approach uses the T and F vectors to visualize the behavior of a normal system user, refer to the blocks “KPI selection” and “Performance blocks” in Figure 3. The selection specifies the type of KPI, it can be a numerical display, a light bulb, a real-time graph, a gauge, etc. Depending on the selection, the design is done in the DT environment and the vectors are either displayed with respect to KPI sections or averaged for an ON/OFF display type. This methodology and its dataflow pipeline are responding to the needs outlined in Section 2.

## 4. Experimentation

**Figure 5:**
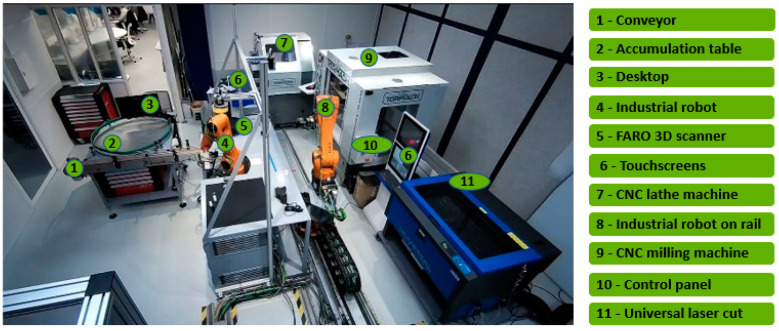
Industry line with system 7 as our focus.

Any framework is subject to important modifications when implemented in an industrial line. Our deployment is executed on a pedagogic manufacturing cell, as seen in Figure 5. Our experimentation is tested on the Computerized Numerical Control (CNC) lathe machine, Tormach 15L SLANT Pro (Manufacturer: Tormach; City: Madison, WI; Country: USA).

### 4.1. Requirements

Manufacturing machines are more complicated to integrate in DT since its sensors are used internally by the controller and the providers only expect that an operator is using it. Nonetheless, Industry 4.0 breaks the myth and puts reality into a future where human operators are replaced with robots for safety measures. In other words, it becomes essential to transform the human-designed machine into CPS for the RAMI 4.0 framework. Analyzing the selected CNC lathe, the embedded sensors are encoders and limit switches, controlling the safety measures, like preventing the machine from over-spinning or deactivating the tool changer if closed spindle or machine extrema. These data are irrelevant to analyze the machine’s performance. So, to gain access to more insights through the DT, IoT sensors are a solution either for local or for cloud deployment. 

**Figure 6:**
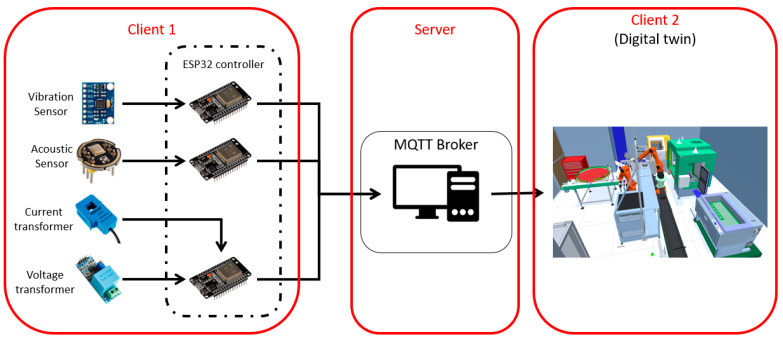
IoT ESP sensor boards, MQTT broker, and Digital Twin.

As described earlier, we are using the Espressif development kit to assemble our IoT sensors. These sensors are built upon our system process. After a discussion with the machinist, we concluded that the vibration, the acoustic emission, and the current are the signals to monitor the process performance. Thus, we developed our IoT sensors based on this conclusion, refer to Figure 6. The acoustic emissions are measured with a microphone and a noise cancellation circuit, the vibration is measured using an accelerometer and a gyroscope, and the current is measured using the power input.

The broker is a service running on any device connected to the network of the IoT sensors and the Digital Twin. In our experimentation, a WPA2-Personal network is used with no subnetting to avoid dealing with networking complexities. The sensors are assembled on three different ESP32 boards with their own controller and installed at corresponding positions: acoustic and vibration sensors are installed near the machining process and the electrical sensor is installed at the power entry.

**Figure 7:**
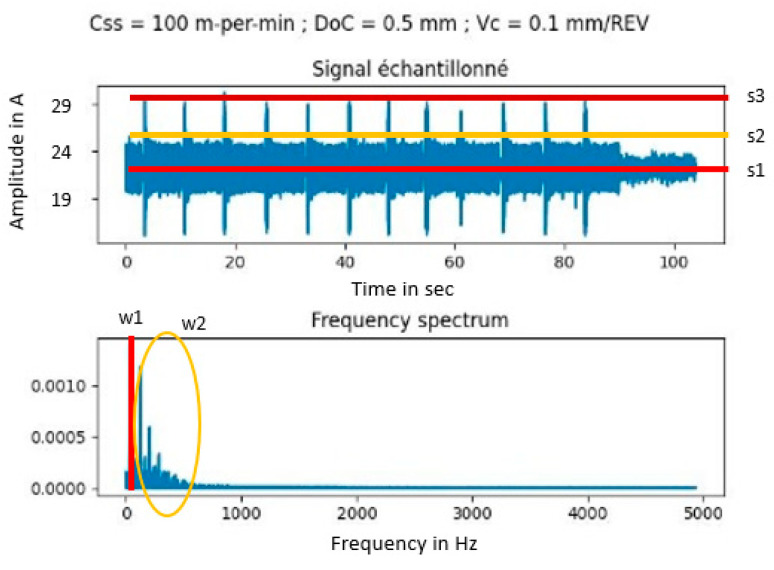
Threshold definitions of maintenance engineer for current evaluation.

Our last requirement is the evaluation of a machine expert for the machine, refer to Figure 7. After discussions, we agreed to set the thresholds of each signal, defining a reference for comparison through arithmetic evaluation. The expert defined five thresholds for each signal $\left(s_{1};s_{2};s_{3};w_{1};w_{2}\right)$, refer to Table 2 for definitions.

The reference report contains a total of 15 thresholds with a tolerance of 5%. Each signal is characterized by five thresholds. We do not introduce the values of reference reports since they are not usable for all the machines. It is always required to let a maintenance engineer evaluate it and attribute these parameters. In our experiment, the signal amplitudes are low since we used a CNC machine to produce plastic parts. 

After all these requirements are set, it is possible for further studies on the mathematical flow in our pipeline to build the KPIs, shown in Figure 4.

### 4.2. Analysis of Signal

For analysis purposes, we explain the analysis and configuration on a small-scale current vector of five samples with a sampling frequency of 1000 Hz. The other signals, acoustic and vibration, follow a similar configuration pipeline. 

We consider five samples of the current signal, shown in Figure 7. This sampling builds our data input with raw values, refer to Equation (3).

(3) $$X_{5,1}={\left[\begin{array}{c} 1648\\ \begin{array}{c} 1196\\ 1647\\ 1194 \end{array}\\ 1635 \end{array}\right]}_{5,1}$$

The input vector is then transformed using Equation (1). However, it is important to find the calibration parameters defined as “$IoT_{max}$” and “$IoT_{min}$”. These values are obtained using an oscilloscope to measure the maximum current, corresponding to the maximum output of ADC, defined as 4095 for a 16-bit converter. Thus, after testing the sensor on different current sources, we obtained that $IoT_{max}=54\;A$ and $IoT_{min}=11\;A$, known that the minimum value for the sensor corresponds when the machine is ON and is not machining. The mapped vector is

(4) $$Y_{5,1}={\left[\begin{array}{c} 28.30\\ \begin{array}{c} 23.55\\ 18.77\\ 23.53 \end{array}\\ 28.16 \end{array}\right]}_{5,1}\left(A\right)$$

The vector in Equation (4) represents a sample from the entire time signal of the current. Then, the FFT is applied to obtain the fundamental frequency $z_{1}$ and the percentage of harmonics $z_{2}$. In our case, we obtain

(5) $$Z_{2,1}={\left[\begin{array}{c} 49.5\\ 0.04 \end{array}\right]}_{2,1}$$

It is essential to note a correlation with the results since in Europe the electric frequency is 50 Hz. In addition, we remark on a small percentage of harmonics caused by the capacitors in the electric board of the CNC machine [18]. Once the vectors Equations (4) and (5) are calculated, the reference thresholds of the reference report are used to obtain the binary vectors for KPI configuration. In our case, the machinist determined the following thresholds:

(6) $$\left(s_{1},s_{2},s_{3},\;w_{1},w_{2}\right)=(21,\;26,\;29.5,\;50,\;0.05)$$

The binary time vector is obtained by multiplying the inverse of $\left(s_{1},s_{2},s_{3}\right)$ with the transpose of Equation (4), obtaining the vector shown in Equation (7).

$$T_{3,5}=\left[\begin{array}{c} \frac{1}{s_{1}}\\ \frac{1}{s_{2}}\\ \frac{1}{s_{3}} \end{array}\right]*Y_{5,1}^{T}=\left[\begin{array}{c} \frac{1}{21}\\ \frac{1}{26}\\ \frac{1}{29.5} \end{array}\right]*\left[\begin{array}{ccc} 28.3 & \begin{array}{ccc} 23.55 & 18.77 & 23.53 \end{array} & 28.16 \end{array}\right]$$

$$T_{3,5}={\left[\begin{array}{c} \begin{array}{ccc} 1.35 & \begin{array}{ccc} 1.12 & 0.89 & 1.12 \end{array} & 1.34 \end{array}\\ \begin{array}{ccc} 1.08 & \begin{array}{ccc} 0.91 & 0.72 & 0.91 \end{array} & 1.08 \end{array}\\ \begin{array}{ccc} 0.95 & \begin{array}{ccc} 0.80 & 0.64 & 0.78 \end{array} & 0.96 \end{array} \end{array}\right]}_{3,5}$$

The proportions of T reveal a normal behavior of the system since the current is oscillating around each set of values, defined by $\left(\frac{average}{s_{1}},\frac{average}{s_{2}},\frac{average}{s_{3}}\right)$. In addition, it is crucial that the values of row 3 in T do not surpass 1. This result shows that the signal is oscillating under the maximum threshold set in the reference report. Before KPI configuration, it is relevant to obtain the frequency properties, denoted by $F_{1,1}$. This vector is obtained by merging the fundamental frequency and the percentage of harmonics.

$$F_{1,1}=\left[\begin{array}{cc} \frac{1}{w_{1}} & \frac{1}{w_{2}} \end{array}\right]*Z_{2,1}=\left[\begin{array}{cc} \frac{1}{50} & \frac{1}{0.05} \end{array}\right]*\left[\begin{array}{c} 49.5\\ 0.04 \end{array}\right]=0.99+0.8=1.79$$

The value of F remains at a constant rate with little to no variations. The ideal value is 2 when the measured fundamental frequency and harmonics percent are equal to the values set in the reference report. To validate the tolerance rate for the value, we discussed it with the electrical team. They used a spectrum analyzer and determined that the acceptable fault, in our case, falls in a range of 8% to 9% for F.

The vectors T and F are conditioned to create the performance indicator inside the 3D environment of DT. It is important to notice that the real input vector is 200 times the length of the one used for clarification in this paper, a size of 1000 samples. The configuration starts by calculating the average of each row in T. The values allow us to understand the signal variation since it should stay constant throughout the process, according to our observations and to the interpretation by machinists. Then, the last row of T is compared, the values above 1 are counted and finally a coefficient is calculated:

(7) $$overpeak=\frac{number\;of\;times\;row_{3}\left(T\right)>1}{number\;of\;columns\left(T\right)}$$

The Equation (7) proportion serves to identify if the signal surpasses a fault, revealing a fault in the process performance. Some other calculations are affected to extract other data for the classification of vector T. However, it is always relevant to compare the values calculated from T with F. If the F-values outbounds the 9% mark, then the machine is certainly in a fault condition either severely or partially. 

These different measurements on T and F are best represented on a gauge, as a type of KPI. In the next section, the classification explanation still considers our five-sample vector of the current signal. 

### 4.3. Configuration of KPI

**Figure 8:**
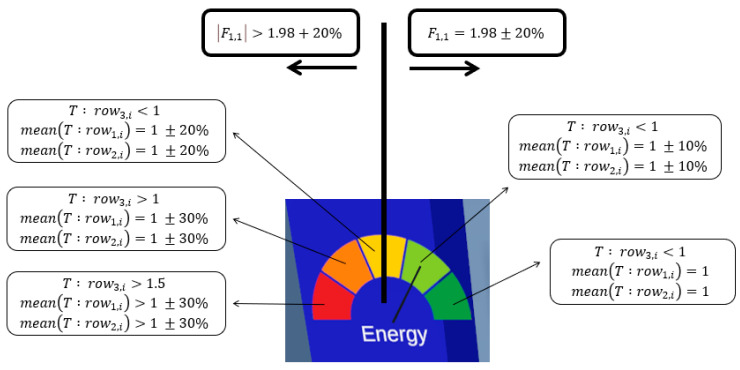
Level gauge for energy monitoring.

According to the calculated values in the previous section, we can determine the number of levels for the gauge to monitor the energy consumption of the CNC machine. The evolution of the current signal represents the energy since it is known to be correlated with the following formula:

$$E=U_{rms}*I_{rms}$$

where, $U_{rms}$ is the root mean square of voltage and $I_{rms}$ is the root mean square of the current. In the AC system, the $U_{rms}$ is at a constant rate of 220-Vac. However, the $I_{rms}$ is subject to change with respect to the process. Thus, the variation of the current time series signal affects the energy consumption of the machine. This energy consumption represents either a fault reflected in the current signal or the consumption due to the machine parameters. For further information, we try to elaborate on the classification of energy-current variation in a five-level gauge, represented in Figure 8.

The bold vertical line represents the boundary between the frequency properties. If $F_{1,1}$ is in good condition, then the arrow falls to the right side; otherwise, it falls to the left side. In addition, the F-vector presents a higher importance than the T-matrix, meaning that even if T fulfills good conditions, refer to the green level in Figure 8, the arrow still points to the left side in the gauge. However, this case is impossible according to the electrical team of our institute.

The $F_{1,1}$ value is assigned to the optimal electrical parameters. In other words, if the current stays at a frequency of 50 Hz and a harmonics percentage of less than 5%, the current that flows in the machine does not present a severe risk. On the other hand, we remarked a correlation with respect to the amplitude of the current, calculated in T-matrix. The third-row value is the maximum amplitude. This threshold defines the operation modes of the electrical components. When the threshold does not surpass a threshold, the electrical system does not overdraw current. However, the slight deviations mean an overdraw and are mostly due to a failure during the process. This deviation is tested, and we remarked when causing a collision during the process, the stepper motors draw more current to push the tool to the right position, causing an overheating of the motor and a deviation in the harmonics of the current signal. This test is realized under the supervision of a professional electrical engineer.

The conditions selected for Gauge configuration are the ones not correlated to other performance data. In our case, we depicted the current signal to monitor the energy, the vibration to monitor the quality of the product, and the acoustic emission to supervise the tool wear. All three KPIs are built the same way, even the conditions are similar as presented in Figure 8 with changes regarding the constants defined. The next section discusses the conditions and responds to the problems found in the literature review of DT.

## 5. Discussion

According to the review of Tao et al. (2019) and Psarommatis et May (2023) presented in Section 2, DT inputs and performance measurements are essential for the building of a successful DT. An additional review paper of 2022 by Botin-Sanabria et al. [19], splits DT into three domains: the Digital Twin Prototype (DTP), the Digital Twin Instance (DTI), and the Performance Digital Twin (PDT). They emphasize that 84 research focuses on DTP and DTI without looking at the performance of DT, validating the problem for DT input identification and performance analysis with respect to the real system.

Our paper introduced an approach to identify the performance data of a physical machine, to assemble and install low-cost effective IoT sensors, and to transform the data into operator readable KPIs, allowing a fast track for possible damages and process drifts. Since the objective is to monitor the CNC lathe performance, we retrieved key positions to place the sensors, especially on the spindle and cutting tool holder. In this position, we retrieved the vibration and acoustic data. In addition, it is possible to connect to the main power supply of the system for current monitoring.

This approach is completed with a detailed data pipeline, explaining the arithmetic framework to transform time series signals into a KPI. Finally, the feasibility is tested by applying it to a CNC lathe machine. Considering the expertise of machinists, the vibration, the current, and the acoustic emissions are considered our main performance data to monitor the operation quality, the energy consumption, and the tool wear. Our results are compatible with the RAMI 4.0 framework since the acquisition, the calculation, and the display time took an average of 105 ms per 1000 samples, making it suitable for implementation on other research projects. Our machine is equipped with three five-level Gauges, representing the degradation of corresponding signals. Red and orange levels cause an emergency stop of the machine, even if the operator acknowledges the error. Thus, we can integrate a virtual emergency stop in our machine, measuring the degradation and acting accordingly. A perspective for performance data fusion is introduced in the conclusion. 

## 6. Conclusions

This paper treats the digitization of a system from the monitoring aspect. With IoT sensors, the existing DT methodologies, proposed in earlier reviews, are augmented by handling performance data and integrating them into the virtual representation. Our novel framework helps the future design of DTs by not only integrating KPIs but also providing the base for process optimization and predictive maintenance of systems. These indicators assist operators in the monitoring of the operations. They require adaption for different systems, but the logical flow of data treatment stays the same. 

At this point, the methodology responds to some key challenges for DT, like “optimization of the cutting parameters of CNC machine”, “state prediction of the CPS”, and “decarbonization of industry” [20]. According to Zezulka et al. [21], these contributions accelerate the deployment of Industry 4.0.

## 7. Perspectives

All proposed frameworks require updates for adaptation either on different machines or on other data types. In our case, the framework can still be augmented by introducing data fusing, by applying it to other machines, such as 3D printers or laser cutters, and by integrating other high-performance algorithms, such as the one of Rosati et al. [22]. From our side, future work is a collaboration with some machinists to put this described correlation upfront to complete the methodology in all aspects of performance monitoring through data fusion.

## Figures and Tables

**Table 1:** Vectors for KPI configuration in DT.

Vector	Value
$X_{n,1}$	Raw data vector on interval n
$Y_{n,1}$	Physical data vector on interval n
$Z_{2,1}$	Frequency properties for input vector
$T_{3,n}$	Binary vector for time series on interval n
$F_{1,1}$	Binary vector for frequency properties

**Table 2:** Definition of machine reference thresholds.

Variable	Meaning
$s_{1}$	It is the average value of the signal
$s_{2}$	It is the upper bound when the machine is not machining
$s_{3}$	It is the upper bound when the machine is machining
$w_{1}$	It is the fundamental frequency during operation
$w_{2}$	It is the percentage of harmonics during operation

## Data Availability

To reproduce our approach, the data and schematics are made available on request and with a confidential agreement between the two parties.
